# Searching for SNPs with cloud computing

**DOI:** 10.1186/gb-2009-10-11-r134

**Published:** 2009-11-20

**Authors:** Ben Langmead, Michael C Schatz, Jimmy Lin, Mihai Pop, Steven L Salzberg

**Affiliations:** 1 Department of Biostatistics, Johns Hopkins Bloomberg School of Public Health, 615 North Wolfe Street, Baltimore, Maryland 21205, USA; 2 Center for Bioinformatics and Computational Biology, University of Maryland, College Park, MD 20742, USA; 3 The iSchool, College of Information Studies, University of Maryland, College Park, MD 20742, USA

## Abstract

As DNA sequencing outpaces improvements in computer speed, there is a critical need to accelerate tasks like alignment and SNP calling. Crossbow is a cloud-computing software tool that combines the aligner Bowtie and the SNP caller SOAPsnp. Executing in parallel using Hadoop, Crossbow analyzes data comprising 38-fold coverage of the human genome in three hours using a 320-CPU cluster rented from a cloud computing service for about $85. Crossbow is available from http://bowtie-bio.sourceforge.net/crossbow/.

## Abstract 2

Novel software utilizing cloud computing technology to cost-effectively align and map SNPs from a human genome in three.

## Rationale

Improvements in DNA sequencing have made sequencing an increasingly valuable tool for the study of human variation and disease. Technologies from Illumina (San Diego, CA, USA), Applied Biosystems (Foster City, CA, USA) and 454 Life Sciences (Branford, CT, USA) have been used to detect genomic variations among humans [1-5], to profile methylation patterns [6], to map DNA-protein interactions [7], and to identify differentially expressed genes and novel splice junctions [8,9]. Meanwhile, technical improvements have greatly decreased the cost and increased the size of sequencing datasets. For example, at the beginning of 2009 a single Illumina instrument was capable of generating 15 to 20 billion bases of sequencing data per run. Illumina has projected [10] that its instrument will generate 90 to 95 billion bases per run by the end of 2009, quintupling its throughput in one year. Another study shows the per-subject cost for whole-human resequencing declining rapidly over the past year [11], which will fuel further adoption. Growth in throughput and adoption are vastly outpacing improvements in computer speed, demanding a level of computational power achievable only via large-scale parallelization.

Two recent projects have leveraged parallelism for whole-genome assembly with short reads. Simpson *et al*. [12] use ABySS to assemble the genome of a human from 42-fold coverage of short reads [2] using a cluster of 168 cores (21 computers), in about 3 days of wall clock time. Jackson and colleagues [13] assembled a *Drosophila melanogaster *genome from simulated short reads on a 512-node BlueGene/L supercomputer in less than 4 hours of total elapsed time. Though these efforts demonstrate the promise of parallelization, they are not widely applicable because they require access to a specific type of hardware resource. No two clusters are exactly alike, so scripts and software designed to run well on one cluster may run poorly or fail entirely on another cluster. Software written for large supercomputers like BlueGene/L is less reusable still, since only select researchers have access to such machines. Lack of reusability also makes it difficult for peers to recreate scientific results obtained using such systems.

An increasingly popular alternative for large-scale computations is cloud computing. Instead of owning and maintaining dedicated hardware, cloud computing offers a 'utility computing' model, that is, the ability to rent and perform computation on standard, commodity computer hardware over the Internet. These rented computers run in a virtualized environment where the user is free to customize the operating system and software installed. Cloud computing also offers a parallel computing framework called MapReduce [14], which was designed by Google to efficiently scale computation to many hundreds or thousands of commodity computers. Hadoop [15] is an open source implementation of MapReduce that is widely used to process very large datasets, including at companies such as Google, Yahoo, Microsoft, IBM, and Amazon. Hadoop programs can run on any cluster where the portable, Java-based Hadoop framework is installed. This may be a local or institutional cluster to which the user has free access, or it may be a cluster rented over the Internet through a utility computing service. In addition to high scalability, the use of both standard software (Hadoop) and standard hardware (utility computing) affords reusability and reproducibility.

The CloudBurst project [16] explored the benefits of using Hadoop as a platform for alignment of short reads. CloudBurst is capable of reporting all alignments for millions of human short reads in minutes, but does not scale well to human resequencing applications involving billions of reads. Whereas CloudBurst aligns about 1 million short reads per minute on a 24-core cluster, a typical human resequencing project generates billions of reads, requiring more than 100 days of cluster time or a much larger cluster. Also, whereas CloudBurst is designed to efficiently discover all valid alignments per read, resequencing applications often ignore or discount evidence from repetitively aligned reads as they tend to confound genotyping. Our goal for this work was to explore whether cloud computing could be profitably applied to the largest problems in comparative genomics. We focus on human resequencing, and single nucleotide polymorphism (SNP) detection specifically, in order to allow comparisons to previous studies.

We present Crossbow, a Hadoop-based software tool that combines the speed of the short read aligner Bowtie [17] with the accuracy of the SNP caller SOAPsnp [18] to perform alignment and SNP detection for multiple whole-human datasets per day. In our experiments, Crossbow aligns and calls SNPs from 38-fold coverage of a Han Chinese male genome [5] in as little as 3 hours (4 hours 30 minutes including transfer time) using a 320-core cluster. SOAPsnp was previously shown to make SNP calls that agree closely with genotyping results obtained with an Illumina 1 M BeadChip assay of the Han Chinese genome [18] when used in conjunction with the short read aligner SOAP [19]. We show that SNPs reported by Crossbow exhibit a level of BeadChip agreement comparable to that achieved in the original SOAPsnp study, but in far less time.

Crossbow is open source software available from the Bowtie website [20]. Crossbow can be run on any cluster with appropriate versions of Hadoop, Bowtie, and SOAPsnp installed. Crossbow is distributed with scripts allowing it to run either on a local cluster or on a cluster rented through Amazon's Elastic Compute Cloud (EC2) [21] utility computing service. Version 0.1.3 of the Crossbow software is also provided as Additional data file 1.

## Results

Crossbow harnesses cloud computing to efficiently and accurately align billions of reads and call SNPs in hours, including for high-coverage whole-human datasets. Within Crossbow, alignment and SNP calling are performed by Bowtie and SOAPsnp, respectively, in a seamless, automatic pipeline. Crossbow can be run on any computer cluster with the prerequisite software installed. The Crossbow package includes scripts that allow the user to run an entire Crossbow session remotely on an Amazon EC2 cluster of any size.

### Resequencing simulated data

To measure Crossbow's accuracy where true SNPs are known, we conducted two experiments using simulated paired-end read data from human chromosomes 22 and X. Results are shown in Tables 1 and 2. For both experiments, 40-fold coverage of 35-bp paired-end reads were simulated from the human reference sequence (National Center for Biotechnology Information (NCBI) 36.3). Quality values and insert lengths were simulated based on empirically observed qualities and inserts in the Wang *et al*. dataset [5].

**Table 1:** Experimental parameters for Crossbow experiments using simulated reads from human chromosomes 22 and X

Reference chromosome	Chromosome 22	Chromosome X
Reference base pairs	49.7 million	155 million
Chromosome copy number	Diploid	Haploid
HapMap SNPs introduced	36,096	71,976
Heterozygous	24,761	0
Homozygous	11,335	71,976
Novel SNPs introduced	10,490	30,243
Heterozygous	6,967	0
Homozygous	3,523	30,243
Simulated coverage	40-fold	40-fold
Read type	35-bp paired	35-bp paired

**Table 2:** SNP calling measurements for Crossbow experiments using simulated reads from human chromosomes 22 and X

	Chromosome 22			Chromosome X		
		
	True number of sites	Crossbow sensitivity	Crossbow precision	True number of sites	Crossbow sensitivity	Crossbow precision
All SNP sites	46,586	99.0%	99.1%	102,219	99.0%	99.6%
Only HapMap SNP sites	36,096	99.8%	99.9%	71,976	99.9%	99.9%
Only novel SNP sites	10,490	96.3%	96.3%	30,243	96.8%	98.8%
						
Only homozygous	14,858	98.7%	99.9%	NA	NA	NA
Only heterozygous	31,728	99.2%	98.8%	NA	NA	NA
						
Only novel het	6,967	96.6%	94.6%	NA	NA	NA
All other	39,619	99.4%	99.9%	NA	NA	NA

Sensitivity is the proportion of true SNPs that were correctly identified. Precision is the proportion of called SNPs that were genuine. NA denotes "not applicable" because of the ploidy of the chromosome.

SOAPsnp can exploit user-supplied information about known SNP loci and allele frequencies to refine its prior probabilities and improve accuracy. Therefore, the read simulator was designed to simulate both known HapMap [22] SNPs and novel SNPs. This mimics resequencing experiments where many SNPs are known but some are novel. Known SNPs were selected at random from actual HapMap alleles for human chromosomes 22 and X. Positions and allele frequencies for known SNPs were calculated according to the same HapMap SNP data used to simulate SNPs.

For these simulated data, Crossbow agrees substantially with the true calls, with greater than 99% precision and sensitivity overall for chromosome 22. Performance for HapMap SNPs is noticeably better than for novel SNPs, owing to SOAPsnp's ability to adjust SNP-calling priors according to known allele frequencies. Performance is similar for homozygous and heterozygous SNPs overall, but novel heterozygous SNPs yielded the worst performance of any other subset studied, with 96.6% sensitivity and 94.6% specificity on chromosome 22. This is as expected, since novel SNPs do not benefit from prior knowledge, and heterozygous SNPs are more difficult than homozygous SNPs to distinguish from the background of sequencing errors.

### Whole-human resequencing

To demonstrate performance on real-world data, we used Crossbow to align and call SNPs from the set of 2.7 billion reads and paired-end reads sequenced from a Han Chinese male by Wang *et al *[5]. Previous work demonstrated that SNPs called from this dataset by a combination of SOAP and SOAPsnp are highly concordant with genotypes called by an Illumina 1 M BeadChip genotyping assay of the same individual [18]. Since Crossbow uses SOAPsnp as its SNP caller, we expected Crossbow to yield very similar, but not identical, output. Differences may occur because: Crossbow uses Bowtie whereas the previous study used SOAP to align the reads; the Crossbow version of SOAPsnp has been modified somewhat to operate within a MapReduce context; in this study, alignments are binned into non-overlapping 2-Mbp partitions rather than into chromosomes prior to being given to SOAPsnp; and the SOAPsnp study used additional filters to remove some additional low confidence SNPs. Despite these differences, Crossbow achieves comparable agreement with the BeadChip assay and at a greatly accelerated rate.

We downloaded 2.66 billion reads from a mirror of the YanHuang site [23]. These reads cover the assembled human genome sequence to 38-fold coverage. They consist of 2.02 billion unpaired reads with sizes ranging from 25 to 44 bp, and 658 million paired-end reads. The most common unpaired read lengths are 35 and 40 bp, comprising 73.0% and 17.4% of unpaired reads, respectively. The most common paired-end read length is 35 bp, comprising 88.8% of all paired-end reads. The distribution of paired-end separation distances is bimodal with peaks in the 120 to 150 bp and 420 to 460 bp ranges.

Table 3 shows a comparison of SNPs called by either of the sequencing-based assays - Crossbow labeled 'CB' and SOAP+SOAPsnp labeled 'SS' - against SNPs obtained with the Illumina 1 M BeadChip assay from the SOAPsnp study [18]. The 'sites covered' column reports the proportion of BeadChip sites covered by a sufficient number of sequencing reads. Sufficient coverage is roughly four reads for diploid chromosomes and two reads for haploid chromosomes (see Materials and methods for more details about how sufficient coverage is determined). The 'Agreed' column shows the proportion of covered BeadChip sites where the BeadChip call equaled the SOAPsnp or Crossbow call. The 'Missed allele' column shows the proportion of covered sites where SOAPsnp or Crossbow called a position as homozygous for one of two heterozygous alleles called by BeadChip at that position. The 'Other disagreement' column shows the proportion of covered sites where the BeadChip call differed from the SOAPsnp/Crossbow in any other way. Definitions of the 'Missed allele' and 'Other disagreement' columns correspond to the definitions of 'false negatives' and 'false positives', respectively, in the SOAPsnp study.

**Table 3:** Coverage and agreement measurements comparing Crossbow (CB) and SOAP/SOAPsnp (SS) to the genotyping results obtained by an Illumina 1 M genotyping assay in the SOAPsnp study

						(SS)		(CB)	
							
Illumina 1 M genotype	Sites	Sites covered (SS)	Sites covered (CB)	Agreed (SS)	Agreed (CB)	Missed allele	Other disagreement	Missed allele	Other disagreement
Chromosome X									
HOM reference	27,196	98.65%	99.83%	99.99%	99.99%	NA	0.004%	NA	0.011%
HOM mutant	10,737	98.49%	99.19%	99.89%	99.85%	NA	0.113%	NA	0.150%
Total	37,933	98.61%	99.65%	99.97%	99.95%	NA	0.035%	NA	0.050%
									
Autosomal									
HOM reference	540,878	99.11%	99.88%	99.96%	99.92%	NA	0.044%	NA	0.078%
HOM mutant	208,436	98.79%	99.28%	99.81%	99.70%	NA	0.194%	NA	0.296%
HET	250,667	94.81%	99.64%	99.61%	99.75%	0.374%	0.017%	0.236%	0.014%
Total	999,981	97.97%	99.70%	99.84%	99.83%	0.091%	0.069%	0.059%	0.108%

'Sites covered' is the proportion of BeadChip sites covered by a sufficient number of sequencing reads (roughly four reads for diploid and two reads for haploid chromosomes). 'Agreed' is the proportion of covered BeadChip sites where the BeadChip call equaled the SOAPsnp/Crossbow call. 'Missed allele' is the proportion of covered sites where SOAPsnp/Crossbow called a position as homozygous for one of two heterozygous alleles called by BeadChip. 'Other disagreement' is the proportion of covered sites where the BeadChip call differed from the SOAPsnp/Crossbow in any other way. NA denotes "not applicable" due to ploidy.

Both Crossbow and SOAP+SOAPsnp exhibit a very high level of agreement with the BeadChip genotype calls. The small differences in number of covered sites (<2% higher for Crossbow) and in percentage agreement (<0.1% lower for Crossbow) are likely due to the SOAPsnp study's use of additional filters to remove some SNPs prior to the agreement calculation, and to differences in alignment policies between SOAP and Bowtie. After filtering, Crossbow reports a total of 3,738,786 SNPs across all autosomal chromosomes and chromosome X, whereas the SNP GFF file available from the YanHaung site [23] reports a total of 3,072,564 SNPs across those chromosomes. This difference is also likely due to the SOAPsnp study's more stringent filtering.

### Cloud performance

The above results were computed on a Hadoop 0.20 cluster with 10 worker nodes located in our laboratory, where it required about 1 day of wall clock time to run. Each node is a four-core 3.2 GHz Intel Xeon (40 cores total) running 64-bit Redhat Enterprise Linux Server 5.3 with 4 GB of physical memory and 366 GB of local storage available for the Hadoop Distributed Filesystem (HDFS) and connected via gigabit ethernet. We also performed this computation using Amazon's EC2 service on clusters of 10, 20 and 40 nodes (80, 160, and 320 cores) running Hadoop 0.20. In each case, the Crossbow pipeline was executed end-to-end using scripts distributed with the Crossbow package. In the 10-, 20- and 40-node experiments, each individual node was an EC2 Extra Large High CPU Instance, that is, a virtualized 64-bit computer with 7 GB of memory and the equivalent of 8 processor cores clocked at approximately 2.5 to 2.8 Ghz. At the time of this writing, the cost of such nodes was $0.68 ($0.76 in Europe) per node per hour.

Before running Crossbow, the short read data must be stored on a filesystem the Hadoop cluster can access. When the Hadoop cluster is rented from Amazon's EC2 service, users will typically upload input data to Amazon's Simple Storage Service (S3) [24], a service for storing large datasets over the Internet. For small datasets, data transfers typically complete very quickly, but for large datasets (for example, more than 100 GB of compressed short read data), transfer time can be significant. An efficient method to copy large datasets to S3 is to first allocate an EC2 cluster of many nodes and have each node transfer a subset of the data from the source to S3 in parallel. Crossbow is distributed with a Hadoop program and driver scripts for performing these bulk parallel copies while also preprocessing the reads into the form required by Crossbow. We used this software to copy 103 gigabytes of compressed short read data from a public FTP server located at the European Bioinformatics Institute in the UK to an S3 repository located in the US in about 1 hour 15 minutes (approximately 187 Mb/s effective transfer rate). The transfer cost approximately $28: about $3.50 ($3.80 in Europe) in cluster rental fees and about $24 ($24 in Europe) in data transfer fees.

Transfer time depends heavily on both the size of the data and the speed of the Internet uplink at the source. Public archives like NCBI and the European Bioinformatics Institute (EBI) have very high-bandwidth uplinks to the >10 Gb/s JANET and Internet2 network backbones, as do many academic institutions. However, even at these institutions, the bandwidth available for a given server or workstation can be considerably less (commonly 100 Mb/s or less). Delays due to slow uplinks can be mitigated by transferring large datasets in stages as reads are generated by the sequencer, rather than all at once.

To measure how the whole-genome Crossbow computation scales, separate experiments were performed using 10, 20 and 40 EC2 Extra Large High CPU nodes. Table 4 presents the wall clock running time and approximate cost for each experiment. The experiment was performed once for each cluster size. The results show that Crossbow is capable of calling SNPs from 38-fold coverage of the human genome in under 3 hours of wall clock time and for about $85 ($96 in Europe).

**Table 4:** Timing and cost for Crossbow experiments using reads from the Wang *et al*. study [5]

EC2 Nodes	1 master, 10 workers	1 master, 20 workers	1 master, 40 workers
Worker CPU cores	80	160	320
Wall clock time	6 h:30 m	4 h:33 m	2 h:53 m
Approximate cluster setup time	18 m	18 m	21 m
Approximate crossbow time	6 h:12 m	4 h:15 m	2 h:32 m
Approximate cost (US/Europe)	$52.36/$60.06	$71.40/$81.90	$83.64/$95.94

Costs are approximate and based on the pricing as of this writing, that is, $0.68 per extra-large high-CPU EC2 node per hour in the US and $0.78 in Europe. Times can vary subject to, for example, congestion and Internet traffic conditions.

**Figure 1:**
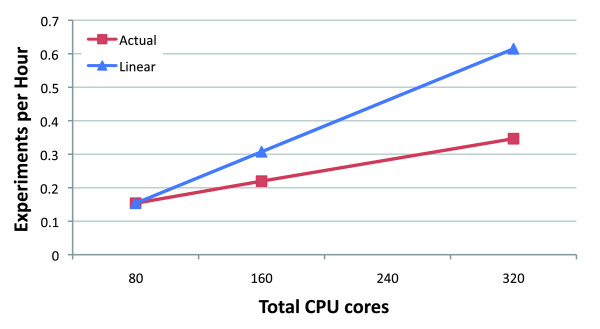
Number of worker CPU cores allocated from EC2 versus throughput measured in experiments per hour: that is, the reciprocal of the wall clock time required to conduct a whole-human experiment on the Wang *et al*. dataset [5]. The line labeled 'linear speedup' traces hypothetical linear speedup relative to the throughput for 80 CPU cores.

Figure 1 illustrates scalability of the computation as a function of the number of processor cores allocated. Units on the vertical axis are the reciprocal of the wall clock time. Whereas wall clock time measures elapsed time, its reciprocal measures throughput - that is, experiments per hour. The straight diagonal line extending from the 80-core point represents hypothetical linear speedup, that is, extrapolated throughput under the assumption that doubling the number of processors also doubles throughput. In practice, parallel algorithms usually exhibit worse-than-linear speedup because portions of the computation are not fully parallel. In the case of Crossbow, deviation from linear speedup is primarily due to load imbalance among CPUs in the map and reduce phases, which can cause a handful of work-intensive 'straggler' tasks to delay progress. The reduce phase can also experience imbalance due to, for example, variation in coverage.

## Materials and methods

### Alignment and SNP calling in Hadoop

Hadoop is an implementation of the MapReduce parallel programming model. Under Hadoop, programs are expressed as a series of map and reduce phases operating on tuples of data. Though not all programs are easily expressed this way, Hadoop programs stand to benefit from services provided by Hadoop. For instance, Hadoop programs need not deal with particulars of how work and data are distributed across the cluster; these details are handled by Hadoop, which automatically partitions, sorts and routes data among computers and processes. Hadoop also provides fault tolerance by partitioning files into chunks and storing them redundantly on the HDFS. When a subtask fails due to hardware or software errors, Hadoop restarts the task automatically, using a cached copy of its input data.

A mapper is a short program that runs during the map phase. A mapper receives a tuple of input data, performs a computation, and outputs zero or more tuples of data. A tuple consists of a key and a value. For example, within Crossbow a read is represented as a tuple where the key is the read's name and the value equals the read's sequence and quality strings. The mapper is generally constrained to be stateless - that is, the content of an output tuple may depend only on the content of the corresponding input tuple, and not on previously observed tuples. This enables MapReduce to safely execute many instances of the mapper in parallel. Similar to a mapper, a reducer is a short program that runs during the reduce phase, but with the added condition that a single instance of the reducer will receive all tuples from the map phase with the same key. In this way, the mappers typically compute partial results, and the reducer finalizes the computation using all the tuples with the same key, and outputs zero or more output tuples. The reducer is also constrained to be stateless - that is, the content of an output tuple may depend only the content of the tuples in the incoming batch, not on any other previously observed input tuples. Between the map and reduce phases, Hadoop automatically executes a sort/shuffle phase that bins and sorts tuples according to primary and secondary keys before passing batches on to reducers. Because mappers and reducers are stateless, and because Hadoop itself handles the sort/shuffle phase, Hadoop has significant freedom in how it distributes parallel chunks of work across the cluster.

The chief insight behind Crossbow is that alignment and SNP calling can be framed as a series of map, sort/shuffle and reduce phases. The map phase is short read alignment where input tuples represent reads and output tuples represent alignments. The sort/shuffle phase bins alignments according to the genomic region ('partition') aligned to. The sort/shuffle phase also sorts alignments along the forward strand of the reference in preparation for consensus calling. The reduce phase calls SNPs for a given partition, where input tuples represent the sorted list of alignments occurring in the partition and output tuples represent SNP calls.

A typical Hadoop program consists of Java classes implementing the mapper and reducer running in parallel on many compute nodes. However, Hadoop also supports a 'streaming' mode of operation whereby the map and reduce functions are delegated to command-line scripts or compiled programs written in any language. In streaming mode, Hadoop executes the streaming programs in parallel on different compute nodes, and passes tuples into and out of the program as tab-delimited lines of text written to the 'standard in' and 'standard out' file handles. This allows Crossbow to reuse existing software for aligning reads and calling SNPs while automatically gaining the scaling benefits of Hadoop. For alignment, Crossbow uses Bowtie [17], which employs a Burrows-Wheeler index [25] based on the full-text minute-space (FM) index [26] to enable fast and memory-efficient alignment of short reads to mammalian genomes.

To report SNPs, Crossbow uses SOAPsnp [18], which combines multiple techniques to provide high-accuracy haploid or diploid consensus calls from short read alignment data. At the core of SOAPsnp is a Bayesian SNP model with configurable prior probabilities. SOAPsnp's priors take into account differences in prevalence between, for example, heterozygous versus homozygous SNPs and SNPs representing transitions versus those representing transversions. SOAPsnp can also use previously discovered SNP loci and allele frequencies to refine priors. Finally, SOAPsnp recalibrates the quality values provided by the sequencer according to a four-dimensional training matrix representing observed error rates among uniquely aligned reads. In a previous study, human genotype calls obtained using the SOAP aligner and SOAPsnp exhibited greater than 99% agreement with genotype calls obtained using an Illumina 1 M BeadChip assay of the same Han Chinese individual [18].

Crossbow's efficiency requires that the three MapReduce phases, map, sort/shuffle and reduce, each be efficient. The map and reduce phases are handled by Bowtie and SOAPsnp, respectively, which have been shown to perform efficiently in the context of human resequencing. But another advantage of Hadoop is that its implementation of the sort/shuffle phase is extremely efficient, even for human resequencing where mappers typically output billions of alignments and hundreds of gigabytes of data to be sorted. Hadoop's file system (HDFS) and intelligent work scheduling make it especially well suited for huge sort tasks, as evidenced by the fact that a 1,460-node Hadoop cluster currently holds the speed record for sorting 1 TB of data on commodity hardware (62 seconds) [27].

### Modifications to existing software

Several new features were added to Bowtie to allow it to operate within Hadoop. A new input format (option --12) was added, allowing Bowtie to recognize the one-read-per-line format produced by the Crossbow preprocessor. New command-line options --mm and --shmem instruct Bowtie to use memory-mapped files or shared memory, respectively, for loading and storing the reference index. These features allow many Bowtie processes, each acting as an independent mapper, to run in parallel on a multi-core computer while sharing a single in-memory image of the reference index. This maximizes alignment throughput when cluster computers contain many CPUs but limited memory. Finally, a Crossbow-specific output format was implemented that encodes an alignment as a tuple where the tuple's key identifies a reference partition and the value describes the alignment. Bowtie detects instances where a reported alignment spans a boundary between two reference partitions, in which case Bowtie outputs a pair of alignment tuples with identical values but different keys, each identifying one of the spanned partitions. These features are enabled via the --partition option, which also sets the reference partition size.

The version of SOAPsnp used in Crossbow was modified to accept alignment records output by modified Bowtie. Speed improvements were also made to SOAPsnp, including an improvement for the case where the input alignments cover only a small interval of a chromosome, as is the case when Crossbow invokes SOAPsnp on a single partition. None of the modifications made to SOAPsnp fundamentally affect how consensus bases or SNPs are called.

### Workflow

**Figure 2:**
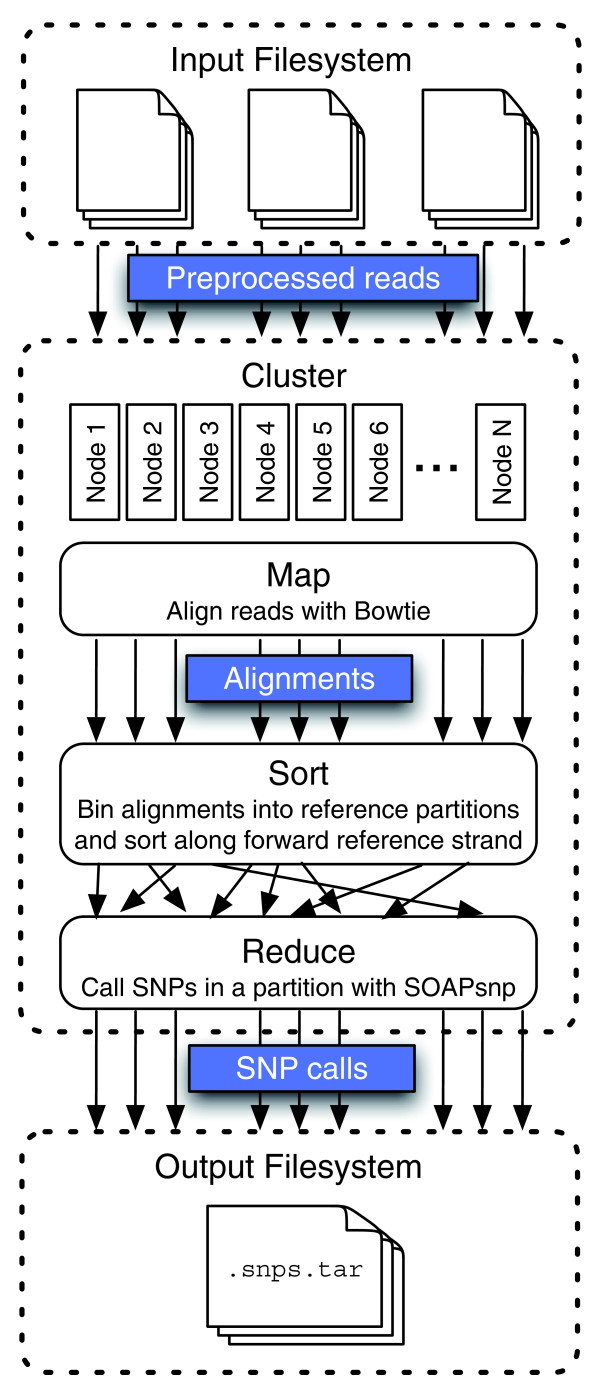
Crossbow workflow. Previously copied and pre-processed read files are downloaded to the cluster, decompressed and aligned using many parallel instances of Bowtie. Hadoop then bins and sorts the alignments according to primary and secondary keys. Sorted alignments falling into each reference partition are then submitted to parallel instances of SOAPsnp. The final output is a stream of SNP calls made by SOAPsnp.

The input to Crossbow is a set of preprocessed read files, where each read is encoded as a tab-delimited tuple. For paired-end reads, both ends are stored on a single line. Conversion takes place as part of a bulk-copy procedure, implemented as a Hadoop program driven by automatic scripts included with Crossbow. Once preprocessed reads are situated on a filesystem accessible to the Hadoop cluster, the Crossbow MapReduce job is invoked (Figure 2). Crossbow's map phase is short read alignment by Bowtie. For fast alignment, Bowtie employs a compact index of the reference sequence, requiring about 3 Gb of memory for the human genome. The index is distributed to all computers in the cluster either via Hadoop's file caching facility or by instructing each node to independently obtain the index from a shared filesystem. The map phase outputs a stream of alignment tuples where each tuple has a primary key containing chromosome and partition identifiers, and a secondary key containing the chromosome offset. The tuple's value contains the aligned sequence and quality values. The soft/shuffle phase, which is handled by Hadoop, uses Hadoop's KeyFieldBasedPartitioner to bin alignments according to the primary key and sort according to the secondary key. This allows separate reference partitions to be processed in parallel by separate reducers. It also ensures that each reducer receives alignments for a given partition in sorted order, a necessary first step for calling SNPs with SOAPsnp.

The reduce phase performs SNP calling using SOAPsnp. A wrapper script performs a separate invocation of the SOAPsnp program per partition. The wrapper also ensures that SOAPsnp is invoked with appropriate options given the ploidy of the reference partition. Files containing known SNP locations and allele frequencies derived from dbSNP [28] are distributed to worker nodes via the same mechanism used to distribute the Bowtie index. The output of the reduce phase is a stream of SNP tuples, which are stored on the cluster's distributed filesystem. The final stage of the Crossbow workflow archives the SNP calls and transfers them from the cluster's distributed filesystem to the local filesystem.

### Cloud support

**Figure 3:**
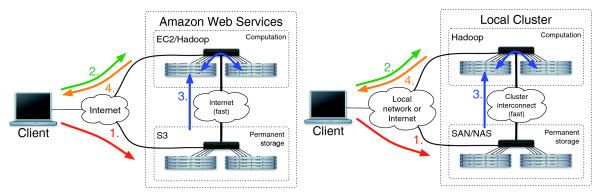
Four basic steps to running the Crossbow computation. Two scenarios are shown: one where Amazon's EC2 and S3 services are used, and one where a local cluster is used. In step 1 (red) short reads are copied to the permanent store. In step 2 (green) the cluster is allocated (may not be necessary for a local cluster) and the scripts driving the computation are uploaded to the master node. In step 3 (blue) the computation is run. The computation download reads from the permanent store, operates on them, and stores the results in the Hadoop distributed filesystem. In step 4 (orange), the results are copied to the client machine and the job completes. SAN (Storage Area Network) and NAS (Network-Attached Storage) are two common ways of sharing filesystems across a local network.

Crossbow comes with scripts that automate the Crossbow pipeline on a local cluster or on the EC2 [21] utility computing service. The EC2 driver script can be run from any Internet-connected computer; however, all the genomic computation is executed remotely. The script runs Crossbow by: allocating an EC2 cluster using the Amazon Web Services tools; uploading the Crossbow program code to the master node; launching Crossbow from the master; downloading the results from the cluster to the local computer; and optionally terminating the cluster, as illustrated in Figure 3. The driver script detects common problems that can occur in the cluster allocation process, including when EC2 cannot provide the requested number of instances due to high demand. The overall process is identical to running on a local dedicated cluster, except cluster nodes are allocated as requested.

### Genotyping experiment

We generated 40-fold coverage of chromosomes 22 and X (NCBI 36.3_ using 35-bp paired-end reads. Quality values were assigned by randomly selecting observed quality strings from a pair of FASTQ files in the Wang *et al*. [5] dataset (080110_EAS51_FC20B21AAXX_L7_YHPE_PE1). The mean and median quality values among those in this subset are 21.4 and 27, respectively, on the Solexa scale. Sequencing errors were simulated at each position at the rate dictated by the quality value at that position. For instance, a position with Solexa quality 30 was changed to a different base with a probability of 1 in 1,000. The three alternative bases were considered equally likely.

Insert lengths were assigned by randomly selecting from a set of observed insert lengths. Observed insert lengths were obtained by aligning a pair of paired-end FASTQ files (the same pair used to simulate the quality values) using Bowtie with options '-X 10000 -v 2 --strata --best -m 1'. The observed mean mate-pair distance and standard deviation for this subset were 422 bp and 68.8 bp, respectively.

Bowtie version 0.10.2 was run with the '-v 2 --best --strata -m 1' to obtain unique alignments with up to two mismatches. We define an alignment as unique if all other alignments for that read have strictly more mismatches. SOAPsnp was run with the rank-sum and binomial tests enabled (-u and -n options, respectively) and with known-SNP refinement enabled (-2 and -s options). Positions and allele frequencies for known SNPs were calculated according to the same HapMap SNP data used to simulate SNPs. SOAPsnp's prior probabilities for novel homozygous and heterozygous SNPs were set to the rates used by the simulator (-r 0.0001 -e 0.0002 for chromosome 22 and -r 0.0002 for chromosome X).

An instance where Crossbow reports a SNP on a diploid portion of the genome was discarded (that is, considered to be homozygous for the reference allele) if it was covered by fewer than four uniquely aligned reads. For a haploid portion, a SNP was discarded if covered by fewer than two uniquely aligned reads. For either diploid or haploid portions, a SNP was discarded if the call quality as reported by SOAPsnp was less than 20.

### Whole-human resequencing experiment

Bowtie version 0.10.2 and a modified version of SOAPsnp 1.02 were used. Both were compiled for 64-bit Linux. Bowtie was run with the '-v 2 --best --strata -m 1' options, mimicking the alignment and reporting modes used in the SOAPsnp study. A modified version of SOAPsnp 1.02 was run with the rank-sum and binomial tests enabled (-u and -n options, respectively) and with known-SNP refinement enabled (-2 and -s options). Positions for known SNPs were calculated according to data in dbSNP [28] versions 128 and 130, and allele frequencies were calculated according to data from the HapMap project [22]. Only positions occurring in dbSNP version 128 were provided to SOAPsnp. This was to avoid biasing the result by including SNPs submitted by Wang *et al*. [5] to dbSNP version 130. SOAPsnp's prior probabilities for novel homozygous and heterozygous SNPs were left at their default values of 0.0005 and 0.001, respectively. Since the subject was male, SOAPsnp was configured to treat autosomal chromosomes as diploid and sex chromosomes as haploid.

To account for base-calling errors and inaccurate quality values reported by the Illumina software pipeline [29,30], SOAPsnp recalibrates quality values according to a four-dimensional matrix recording observed error rates. Rates are calculated across a large space of parameters, the dimensions of which include sequencing cycle, reported quality value, reference allele and subject allele. In the previous study, separate recalibration matrices were trained for each human chromosome; that is, a given chromosome's matrix was trained using all reads aligning uniquely to that chromosome. In this study, each chromosome is divided into non-overlapping stretches of 2 million bases and a separate matrix is trained and used for each partition. Thus, each recalibration matrix receives less training data than if matrices were trained per-chromosome. Though the results indicate that this does not affect accuracy significantly, future work for Crossbow includes merging recalibration matrices for partitions within a chromosome prior to genotyping.

An instance where Crossbow reports a SNP on a diploid portion of the genome is discarded (that is, considered to be homozygous for the reference allele) if it is covered by fewer than four unique alignments. For a haploid portion, a SNP is discarded if covered by fewer than two unique alignments. For either diploid or haploid portions, a SNP is discarded if the call quality as reported by SOAPsnp is less than 20. Note that the SOAPsnp study applies additional filters to discard SNPs at positions that, for example, are not covered by any paired-end reads or appear to have a high copy number. Adding such filters to Crossbow is future work.

## Discussion

In this paper we have demonstrated that cloud computing realized by MapReduce and Hadoop can be leveraged to efficiently parallelize existing serial implementations of sequence alignment and genotyping algorithms. This combination allows large datasets of DNA sequences to be analyzed rapidly without sacrificing accuracy or requiring extensive software engineering efforts to parallelize the computation.

We describe the implementation of an efficient whole-genome genotyping tool, Crossbow, that combines two previously published software tools: the sequence aligner Bowtie and the SNP caller SOAPsnp. Crossbow achieves at least 98.9% accuracy on simulated datasets of individual chromosomes, and better than 99.8% concordance with the Illumina 1 M BeadChip assay of a sequenced individual. These accuracies are comparable to those achieved in the prior SOAPsnp study once filtering stringencies are taken into account.

When run on conventional computers, a deep-coverage human resequencing project requires weeks of time to analyze on a single computer by contrast, Crossbow aligns and calls SNPs from the same dataset in less than 3 hours on a 320-core cluster. By taking advantage of commodity processors available via cloud computing services, Crossbow condenses over 1,000 hours of computation into a few hours without requiring the user to own or operate a computer cluster. In addition, running on standard software (Hadoop) and hardware (EC2 instances) makes it easier for other researchers to reproduce our results or execute their own analysis with Crossbow.

Crossbow scales well to large clusters by leveraging Hadoop and the established, fast Bowtie and SOAPsnp algorithms with limited modifications. The ultrafast Bowtie alignment algorithm, utilizing a quality-directed best-first-search of the FM index, is especially important to the overall performance of Crossbow relative to CloudBurst. Crossbow's alignment stage vastly outperforms the fixed-seed seed-and-extend search algorithm of CloudBurst on clusters of the same size. We expect that the Crossbow infrastructure will serve as a foundation for bringing massive scalability to other high-volume sequencing experiments, such as RNA-seq and ChIP-seq. In our experiments, we demonstrated that Crossbow works equally well either on a local cluster or a remote cluster, but in the future we expect that utility computing services will make cloud computing applications widely available to any researcher.

## Abbreviations

EC2: Elastic Compute Cloud; FM: full-text minute-space; HDFS: Hadoop Distributed Filesystem; NCBI: National Center for Biotechnology Information; S3: Simple Storage Service; SNP: single nucleotide polymorphism.

## Authors' contributions

BL and MCS developed the algorithms, collected results, and wrote the software. JL contributed to discussions on algorithms. BL, MCS, JL, MP, and SLS wrote the manuscript.

## Additional data files

The following additional data are included with the online version of this article: version 0.1.3 of the Crossbow software (Additional data file 1).

## Supplementary Material

Additional data file 1 Version 0.1.3 of the Crossbow software. Click here for file
